# Spatial Analysis of Prostate Cancer in Texas Counties

**DOI:** 10.1093/geroni/igaa057.483

**Published:** 2020-12-16

**Authors:** Ganesh Baniya, Joseph Oppong

**Affiliations:** University of North Texas, Denton, Texas, United States

## Abstract

Prostate cancer (PCa) is the most common cancer in American men, with estimated 191,930 new cases and about 33,330 deaths for 2020 (American Cancer Society (ACS), 2020). In Texas, there were 10,660 new cases and 1,900 deaths from prostate cancer in 2019 and only about 53.6% of men had ever talked to a healthcare professional about the advantages of the PSA test (Texas Department of State Health Services, 2018). The incidence of PCa is about 60% higher in blacks than in whites for unknown reasons (ACS, 2019), and once cancer has reached to its latent state, other comorbidities such as low sexual satisfaction, urinary incontinence and obstruction, and bowel dysfunction can decrease the quality of life even after surgery. A four-fold, worldwide increase in males above 65 years is expected by 2050 (Ferlay et al., 2010). Thus, with an increasing elderly population, the percentage of men needing new diagnoses and effective treatment will also rise. Previous studies have identified increasing age, African ancestry, a family history of the disease, and certain inherited genetic conditions as significant risk factors for prostate cancer (ACS, 2019). This study examined the geography and change at county-level PCa mortality rates in Texas from 1999-2009 using Texas vital web dataset in relation to smoking, race/ethnicity, obesity, alcohol consumption, and insurance coverage. Results showed that PCa is concentrated mostly in the western, eastern and central parts of TX and is positively correlated with smoking, obesity, PCP per 100,000 and negatively related to alcohol consumption and percent uninsured.

